# An Aptamer-Based Biosensor for Colorimetric Detection of *Escherichia coli* O157:H7

**DOI:** 10.1371/journal.pone.0048999

**Published:** 2012-11-07

**Authors:** Wenhe Wu, Jie Zhang, Meiqin Zheng, Yuhong Zhong, Jie Yang, Yuhong Zhao, Wenping Wu, Wei Ye, Jie Wen, Qi Wang, Jianxin Lu

**Affiliations:** 1 Key Laboratory of Laboratory Medicine, Ministry of Education, Zhejiang Provincial Key Laboratory of Medical Genetics, Wenzhou Medical College, Wenzhou, Zhejiang, People’s Republic of China; 2 Department of Clinical Laboratory Medicine, The Affiliated Eye Hospital of Wenzhou Medical College, Wenzhou, People’s Republic of China; 3 Department of Respiratory Medicine, The Second Hospital Affiliated to Dalian Medical University, Dalian, People’s Republic of China; University of Houston, United States of America

## Abstract

**Background:**

An aptamer based biosensor (aptasensor) was developed and evaluated for rapid colorimetric detection of *Escherichia coli* (*E*. *coli*) O157:H7.

**Methodology/Principal Findings:**

The aptasensor was assembled by modifying the truncated lipopolysaccharides (LPS)-binding aptamer on the surface of nanoscale polydiacetylene (PDA) vesicle using peptide bonding between the carboxyl group of the vesicle and the amine group of the aptamer. Molecular recognition between *E. coli* O157:H7 and aptamer at the interface of the vesicle lead to blue-red transition of PDA which was readily visible to the naked eyes and could be quantified by colorimetric responses (CR). Confocal laser scanning microscope (CLSM) and transmission electron microscopy (TEM) was used to confirm the specific interactions between the truncated aptamer and *E*. *coli* O157:H7. The aptasensor could detect cellular concentrations in a range of 10^4^∼ 10^8^ colony-forming units (CFU)/ml within 2 hours and its specificity was 100% for detection of *E. coli* O157:H7. Compared with the standard culture method, the correspondent rate was 98.5% for the detection of *E*. *coli* O157:H7 on 203 clinical fecal specimens with our aptasensor.

**Conclusions:**

The new aptasensor represents a significant advancement in detection capabilities based on the combination of nucleic acid aptamer with PDA vesicle, and offers a specific and convenient screening method for the detection of pathogenic bacteria. This technic could also be applied in areas from clinical analysis to biological terrorism defense, especially in low-resource settings.

## Introduction

In recent years, outbreaks of foodborne diseases associated with pathogenic *Escherichia Coli* (*E*. *coli*) have been widely spread and grown as *public health problems*. Most of what we know about pathogenic *E. coli* comes from the outbreak studies of *E. coli* O157:H7 infection, which was first identified as a pathogen in 1982 [1]. According to the Centers for Disease Control and Prevention (CDC), *E*. *coli* O157:H7 infections accounted for 32 outbreaks between 2003 and 2008 in United States [2] including large outbreaks related to contaminated spinach, iceberg and romaine lettuce [3], [4]. The *E*. *coli* O157:H7 is a specific serotype of *E. coli*, which can cause watery diarrhea, hemorrhagic colitis, hemolytic-uremia syndrome (HUS) and thrombotic thrombocytopenic purpura (TTP), especially in young children and the elderly. The conventional assays to detect *E*. *coli* O157:H7 are specimen culture and colony counting which are sensitive and selective enough. But, these methods are labor intensive, time consuming (requiring 5 to 7 days) and professional operation limited. Many types of *E*. *coli* O157:H7 rapid detection tests have been developed during the last decades, involving nucleic acid-based polymerase chain reaction (PCR) technology [5], [6], enzyme-linked immunosorbent assay (ELISA) [7] and immunomagnetic separation method [8] and microarray technology [9]. However, PCR-based assay and microarray technology has high risk of false result owing to inhibition by components of the sample matrix, and ELISA requires extra labeled antibodies, while immunomagnetic separation method often requires a combination of other methods.

Various kinds of biosensors have been also developed to detect the *E*. *coli* O157:H7, including microarray biosensor [10], [11], immunosensor [12], [13], surface plasmon resonance (SPR) biosensor [14], [15], waveguide biosensor [16] and so on. But these test results can not be easily seen by naked eyes due to professional instrument and operation limited. Additionally, *the antibody* disadvantages *associated with their production*, *stability* and crossreaction have prompted us to seek alternatives of molecular recognition component. Other biosensors, which are based on oligonucleotide [17], have been also proposed. But they often require short enrichment or DNA extraction. Thus, the developments of improved and easy methods are emergent, especially those primary screening tests of the outbreak of the foodborne diseases.

Due to their unique optical properties, polydiacetylene (PDA) and its derivatives have been widely exploited for biosensing applications [18], [19]. Our research group and others [20], [21] have found that diacetylenes could undergo polymerization via 1, 4-addition reaction and form alternating eneyne polymer chains by ultravioletb (UV) irradiation at room temperature. The resulting polymer showed a maximum absorbance around 640 nm (blue form) in the visible region of the spectrum. Particularly, the maximum absorbance changed from 640 nm to 540 nm and the color changed through violet from blue to red in response to external stimuli, such as temperature [22], pH change [23], surface pressure [24] and molecular recognition [25], [26].

Nucleic acid aptamers are relatively short single-stranded DNA or RNA oligonucleotides that have been engineered through repeated rounds of *in vitro selection* referred to as systematic evolution of ligands by exponential enrichment (SELEX) [27], [28]. They are created to bind to specific targets such as small molecules, proteins, nucleic acids, and even cells, tissues and organisms [29]. Owing to their vitro selection, high affinity and specificity, aptamers are beginning to emerge as new molecular recognition components that rival antibodies in biosensing applications [30].

**Figure 1:**
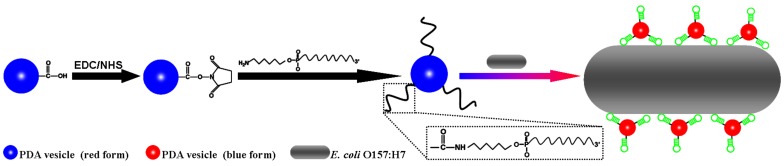
Principle of *E. coli* O157:H7 detection using aptasensor.

In this paper, an aptamer based biosensor (aptasensor) was developed and evaluated for rapid colorimetric detection of *E. coli* O157:H7. 10, 12-pentacosadiynoic acid (PCDA), one of the well-known diacetylene monomers, was used to prepare PDA vesicles. It consists of hydrophillic head group and hydrophobic tail, can be easily self-assembled into vesicles in solution. The free carboxyl of nanoscale PDA vesicle was activated with N-hydroxysuccinimide (NHS) and 1-Ethyl-3-(3-dimethylaminopropyl)carbodiimide (EDC), and formed reactive intermediate. The aptasensor was obtained by modifying the truncated 5′-amino-aptamer on the surface of nanoscale PDA vesicle using peptide bonding between the carboxyl group of the vesicle and the amine group of the aptamer (Figure 1). Molecular recognition between *E. coli* O157:H7 and aptamer at the interface of the vesicle lead to blue-red transition of PDA vesicle which was readily visible to the naked eyes and could be quantified by colorimetric responses (CR).

## Results and Discussion

### Aptamers Modified and Conjugation

**Figure 2:**
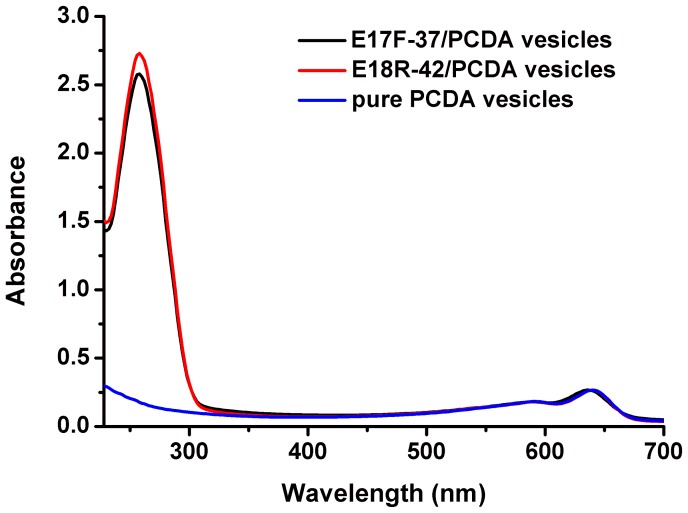
UV-vis absorbance spectra of pure PCDA vesicles before (blue curve) and after binding aptamers (red and black curve).

Unpolymerized vesicles were made by using PCDA, DMPC/PCDA or LPS/DMPC/PCDA in the dark room. Then the PDA vesicles were polymerized via 1, 4-addition reaction to form alternating polymer chains under UV light irratidiation at 254 nm. The resulting polyers appeared deeply blue which is directly visible to the unaided eye and can be characterized by the UV-vis spectrum showing an absorption peak at 640 nm (Figure 2, blue curve). After binding the aptamers (E17F-37 and E18R-42) to the PDA vesicles, the absorption peak at 640 nm did not change, and an additional absorption peak appeared at 260 nm (characteristic absorption peak of the nucleotide), which meant that the aptamers had been modified to PDA vesicles (Figure2, black and red curve). By detecting the optical absorbance of the samples at 260 nm before and after dialysis, the efficiency of conjugation was about 85%. It was estimated that aptamer conjugated on pure PCDA vesicle of 50 nmol (18.73 mg) could reach 4.25 nmol. The pure PCDA vesicles, aptamer/PCDA vesicles, DMPC/PCDA vesicles and LPS/DMPC/PCDA vesicles, confirmed by transmission electron microscopy (TEM), were produced successfully. Four kinds of PDA vesicles appeared to be nearly monodispersed, with diameters of 30–100 nm (shown in Figure S1).

### CLSM Images of Affinity Tests

To address the effect of affinity between aptamers and *E*. *coli* O157:H7, we prepared fluorescein isothiocyanate (FITC) labeled aptamers before and after truncated. Positive controls were investigated with FITC labeled E17F-72 (or E18R-72) instead of FITC labeled E17F-37 (or E18R-42). Further, *Salmonella typhimurium* was used instead of *E*. *coli* O157:H7 in the experiment as negative controls. Figure S2a, b, d and e shows representative CLSM images of *E*. *coli* O157:H7 after incubating of FITC labeled E17F-37, E18R-42, E17F-72and E18R-72 separately. Green fluorescence was detected on *E*. *coli* O157:H7 with all four kinds of FITC labeled aptamers. It was well known that the recognition and binding between the aptamers and target molecules were depended on their structures, like the interactions between antigen and antibody, the results also might indicate that the aptamers before and after truncated had the same loops structures and similar functions. Moreover, no green fluorescence was detected on *Salmonella typhimurium* with FITC labeled E17F-37, E18R-42 (Figure S2c and f). It provided preliminary evidence of specificity of aptamers after truncated.

### TEM Images of Affinity Tests

**Figure 3:**
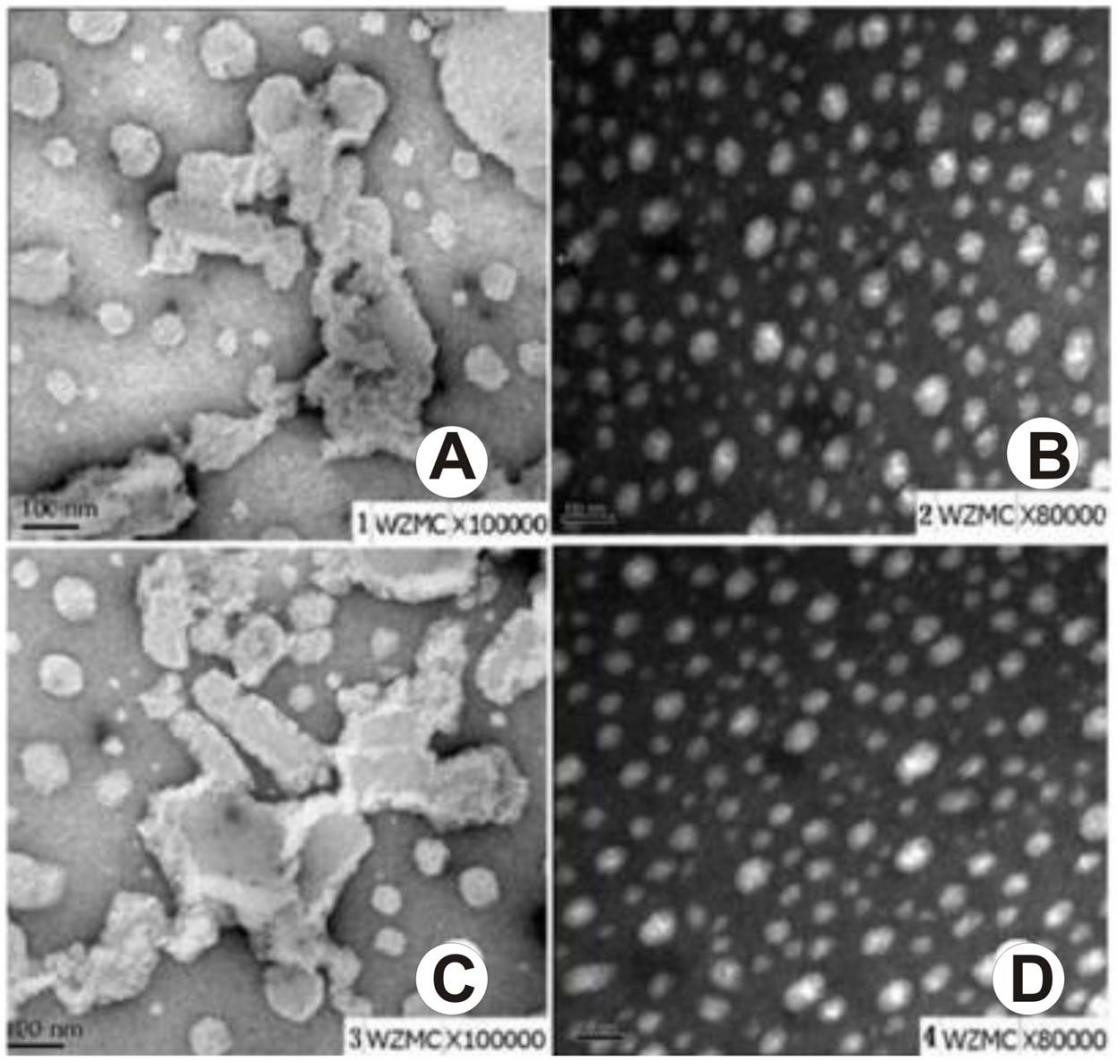
Negative-stained TEM images. (A) E17F-37/PCDA vesicles with LPS/DMPC/PCDA. (B) E17F-37/PCDA vesicles with DMPC/PCDA. (C) E18R-42/PCDA vesicles with LPS/DMPC/PCDA. (D) E18R-42/PCDA vesicles with DMPC/PCDA. Scale bar is 100 nm.

In order to elucidate the mechanism behind the aptasensors in recognition, we generated the LPS/DMPC/PCDA vesicles to simulate the *E. coli* O157:H7. Because of high hydrophobicity of the lipid A in LPS, LPS could insert into vesicles easily by the hydrophobic interaction between DMPC and lipid A, leaving the polysaccharide free on the surface of vesicles. The aptamer/PCDA vesicles were added to the LPS/DMPC/PCDA vesicles and the DMPC/PCDA vesicles, the results were observed by TEM. As shown in Figure 3, after adding the aptamer/PCDA vesicles to the DMPC/PCDA vesicles, we observed that all the aptasensors were spherical in shape with an approximate diameter of less than 100 nm, with no change in the morphology and being separated well. However, after adding the aptamer/PCDA vesicles to the LPS/DMPC/PCDA vesicles, there were are dramatically changes in their morphology, leading to the formation of aggregate clusters with size that over 100 nm. These results also confirmed that the aptasensors could specifically recognize the LPS of *E*. *coli* O157:H7, suggesting that the aptasensors might respond to LPS in *E*. *coli* O157:H7 and could be used for detecting *Escherichia coli* as well.

### Colorimetric Detection of *E. coli* O157:H7 with the Aptasensor

**Figure 4:**
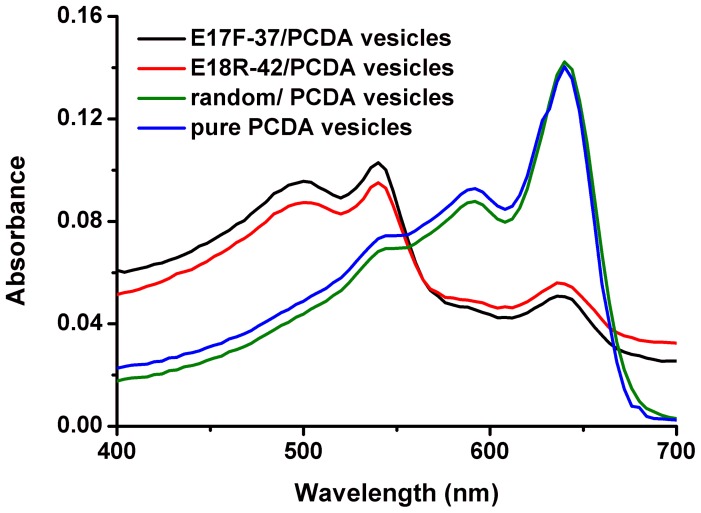
Representative spectrum for *E*. *coli* O157:H7 (10^8^CFU/ml) in presence of E17F-37/PCDA vesicles (black curve), E18R-42/PCDA vesicles (red curve), random/PCDA vesicles (green curve) and pure PCDA vesicles (blue curve).

**Figure 5:**
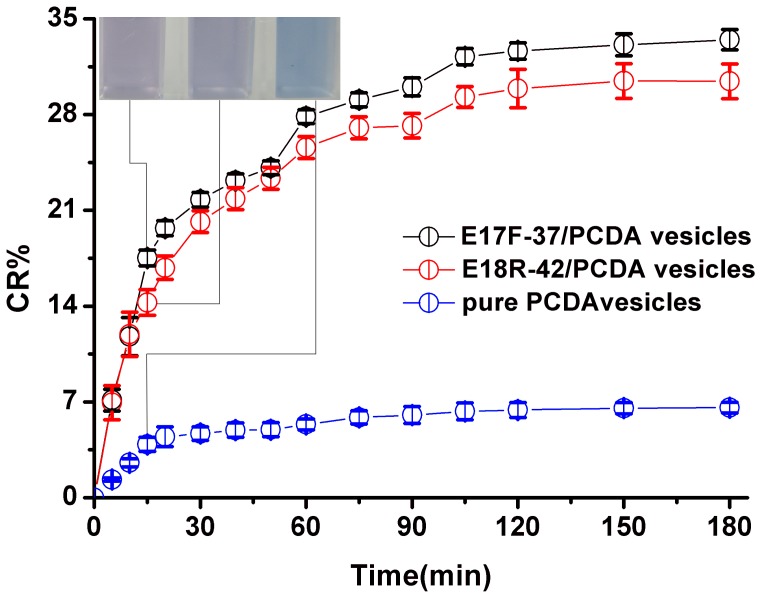
Kinetic study for the CR% change of *E*. *coli O*157:H7 with E17F-37/PCDA vesicles (black curve), E18R-42/PCDA vesicles (red curve) and pure PCDA vesicles (blue curve). Inset: Visual detection in 15 minutes.

The aptasensors appeared deeply blue and showed an absorption maximum at 640 nm. After being incubated with *E*. *coli* O157:H7 solution (50 µl 10^8^ colony-forming units (CFU)/ml) at 37°C temperature under stirring, the color of aptasensors turned from deeply blue to red, which could be easily seen by naked eyes. In contrast to pure PCDA vesicles (Figure 4, blue curve) and random/PCDA vesicles (Figure 4, green curve), the absorption maximum shifted from 640 to 540 nm,and a new absorption (500 nm) appeared after the aptasensors (E17F-37/PCDA vesicles, E18R-42/PCDA vesicles) were exposed to *E*. *coli* O157:H7 (Figure 4, black curve and red curve). All this indicated that the color changes might be caused by the reaction between aptamers and *E. coli* O157:H7. In order to further validate whether the color change was resulted from the conjugation of the aptasensor and the bacterium, we added the *E*. *coli* O157:H7 with a concentration of 10^7^CFU/ml to the aptasensors (E17F-37/PCDA vesicles, E18R-42/PCDA vesicles) and pure PCDA vesicles, and drew the CR%-time curve. As shown in Figure 5, as the time went on, the CR% values gradually increased. Both aptasensors reacted with bacteria quickly (Figure 5, black curve and red curve). In 15 minutes, both CR%s were almost 15%, and reached saturation in almost 120 min. However, for the control (Figure 5, blue curve), no color change was observed by naked eye and the CR% was less than 7%. Thus, these results suggested that the color changes were resulted from the reaction between aptamers and *E*. *coli* O157:H7.

### Sensitivity Analysis and Linear Range

**Figure 6:**
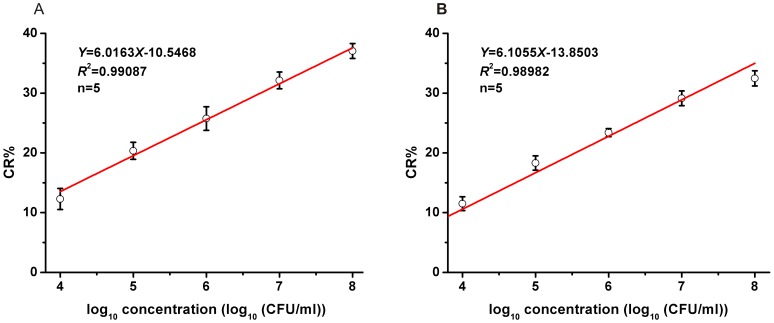
Calibration curve for CR% vs. concentration of *E*. *coli O*157:H7 using by different PDA vesicles. (A) CR% of E17F-37/PCDA vesicles with *E. coli O*157:H7. (B) CR% of E18R-42/PCDA vesicles with *E. coli O*157:H7.

To quantify the sensitivity of the aptasensors, we challenged with an increasing concentration of bacteria and generated a calibration curvet. As shown in Figure 6, when the concentration of bacteria increased, the value of CR% enhanced as well, which meant, with the increasing of the concentration of bacteria, the tendency of the color changed from blue to red, and the CR%s increased gradually.

Also, a good linear relationship was shown when the concentrations of bacteria was in the range of 10^4^ ∼ 10^8^CFU/ml. Figure 6 (A) showed the linear the linear equation for the reaction between the E17F-42/PCDA vesicles and the *E*. *coli* O157:H7 was *Y* = 6.0163*X*-10.5468 (*R* ^2^ = 0.99087), the detect limitation was 10^4^ CFU/ml (three times the standard deviation of blank). The Figure 6 (B) showed the linear equation for the reaction between the E18R-42/PCDA vesicles and the *E*. *coli* O157:H7: the linear equation was *Y* = 6.1055*X*-13.8503 (*R* ^2^ = 0.98982) and the detect limitation was 10^4^ CFU/ml too, These detection limits were 10^4^-fold lower than previously reported PDA-based approach [31] and 10^2^-fold lower [12] than or comparable [11], [15] to the presently available immunosensor (see performance comparison in Table 1).

**Table 1:** Performance comparison between biosensors for the Detection of *E. coli* O157:H7.

Type	LOD (CFU/ml)	Visible to the naked eyes	Enrichment or DNA extraction	Molecular recognition component	Ref
microelectrode array biosensor	10^7^	No	No	antibody	[10]
electrochemical impedance immunosensor	10^6^	No	No	antibody	[12]
SPR immunosensor	3.0 × 10^4^	No	No	antibody	[15]
Chemiluminescence microarray readout system	10^4^	No	No	antibody	[11]
piezoelectric immunosensor	10^3^	No	No	antibody	[13]
SAM based SPR biosensor	10^3^	No	No	antibody	[14]
waveguide biosensor	10^3^	No	Yes	antibody	[16]
QCM DNA sensor	2.67×10^2^	No	Yes	oligonucleotide	[17]
**PDA-based aptasensor**	**10^4^**	**Yes**	**No**	**oligonucleotide**	**This study**

### Specificity Analysis

The recognition of aptamer to target molecule was highly specific which could identify the target accurately from similar molecules with subtle difference. In order to confirm that the color shift was specifically caused by *E*. *coli O*157:H7, the aptasensors were challenged with other types of bacteria including *E. coli* (ATCC 25922, CMCC44825, CMCC44155, CMCC44151), *Salmonella typhimurium*, *Salmonella typhi, Salmonella paratyphi* A, *Salmonella paratyphi* B, *Shigella sonnei*, *Shigella flexneri*, *Proteus vulgaris*, *Enterobacter aerogenes*, *Citrobacter freundii*, *Staphylococcus aureus* and *Monilia albican.*

**Figure 7:**
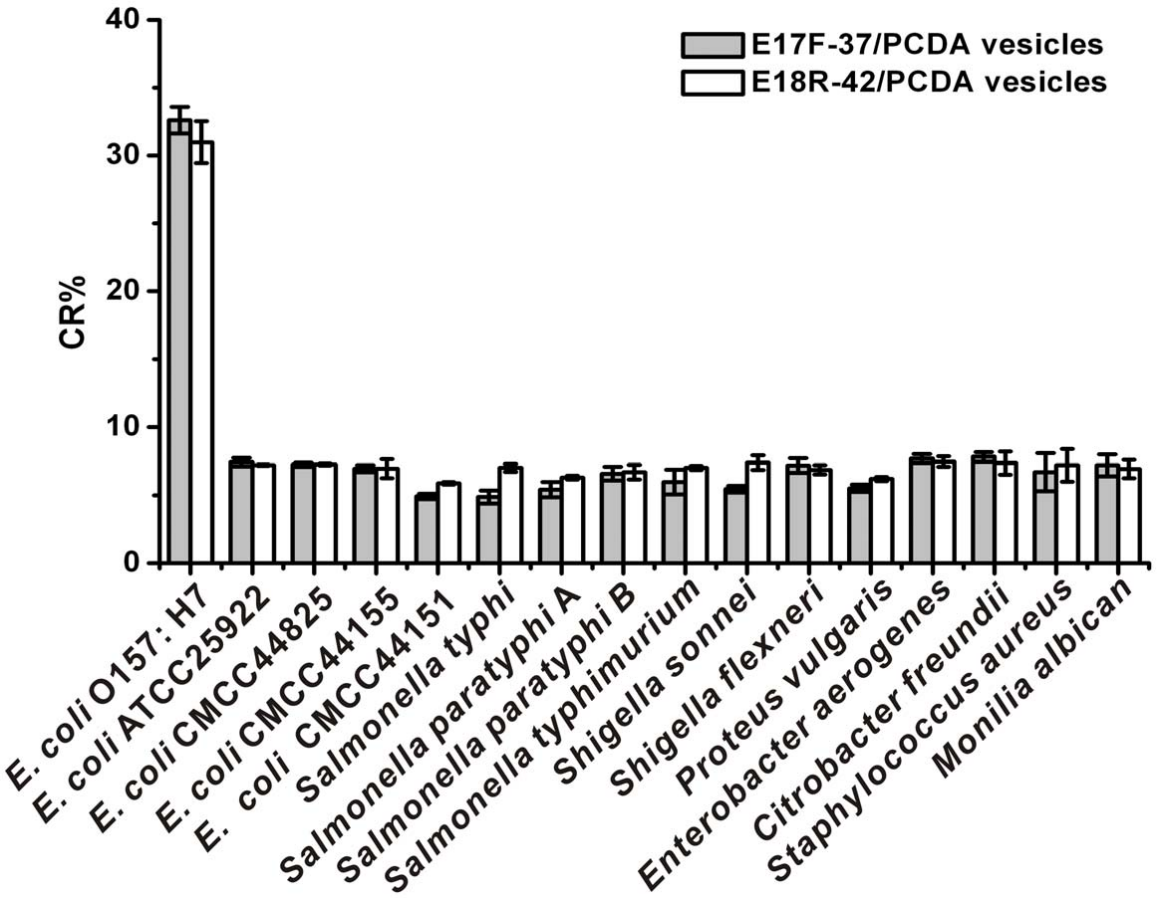
Specificity of aptamer-based biosensor detection *E. coli* O157: H7 (10^7^CFU/ml *E*. *coli* O157:H7, all other bacteria of 10^8^CFU/ml).

*E*. *coli O*157:H7 was diluted to the concentration of 10^7^CFU/ml while other bacteria were diluted to the concentration of 10^8^CFU/ml by deionized water, and then be incubated with aptasensors for 2 hours. The values of CR%s were calculated as previously mentioned. As shown in Figure 7, only the *E*. *coli* O157:H7 could be recognized by the aptasensors, which means our biosensors could only specially detect the *E*. *coli* O157:H7. Importantly, this selectivity can be visualized with the naked eye.

Indeed, only the aptasensors containing *E*. *coli* O157:H7 were red, whereas all others (10-fold higher than *E*. *coli O*157:H7) have remained blue. This clearly illustrated that the aptasensors was only specific to the *E*. *coli* O157:H7 and did not respond to the wide range of background bacteria even to other *E*. *coli.*

### Detection of *E. coli* O157:H7 in Fecal Samples

**Figure 8:**
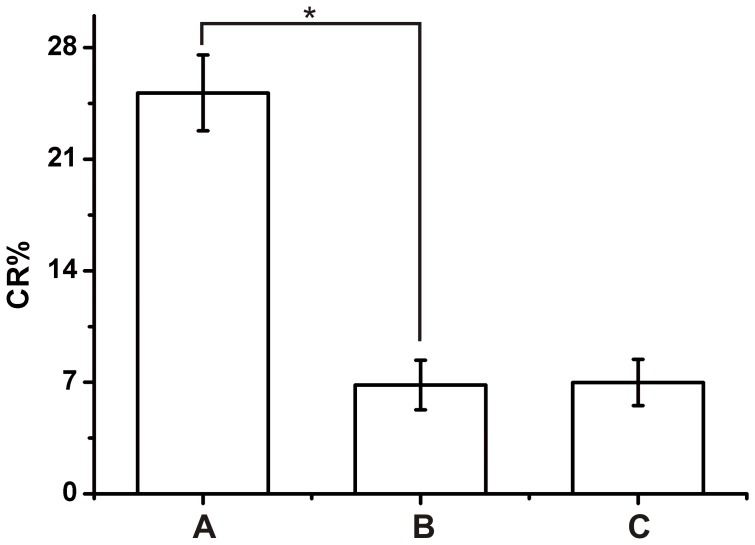
Application of E18R-42/PCDA vesicles in fecal samples contaminated artificially (10^8^ CFU/ml initial concentration of *E*. *coli* O157:H7). (A) CR% of E18R-42/PCDA vesicles with artificially contaminated fecal samples. (B) CR% of E18R-42/PCDA vesicles with uncontaminated fecal samples. (C) CR% of pure PCDA vesicles with artificially contaminated fecal samples. *, *p*<0.05 by Student’s *t* test.

The 100 fecal samples artificially contaminated were exposed to E18R-42/PCDA vesicles and pure PCDA vesicles for 2 hours. As shown in Figure 8, group A (E18R-42/PCDA vesicles with artificially contaminated fecal samples) produced 3 times the CR% of group B (E18R-42/PCDA vesicles with uncontaminated fecal samples) and group C (pure PCDA vesicles with artificially contaminated fecal samples). The CR%s difference between these groups demonstrated that this sensor could determine *E*. *coli O*157:H7 in artificially contaminated fecal samples and It is feasible to separate *E*. *coli* O157:H7 as pretreatment process. However, the pretreatment process inevitably decreased the concentration of *E*. *coli O*157:H7 and the CR% of group A were less than those of E18R-42/PCDA vesicles with *E*. *coli O*157:H7 (10^8^ CFU/ml). Furthermore, in order to quantitatively compare the difference responses of E18R-42/PCDA vesicles to the artificially contaminated and uncontaminated fecal samples, a Student’s t-test was conducted. Based on Figure 8, the responses of the E18R-42/PCDA vesicles to the 100 artificially contaminated and uncontaminated fecal samples showed a statistically significant difference.

In addition, the clinical fecal specimens (*n* = 203) were tested for detecting *E*. *coli* O157:H7 by standard culture method and our E18R-42/PCDA vesicles. Samples were deemed to be aptasensor – positive if CR%s were equal or greater than 10%. All samples resulted in 98.5% (200 to 203) agreement of *E*. *coli* O157:H7 detection between standard culture method and our E18R-42/PCDA vesicles. The agreement ratewas the proportion of all samples diagnosed correctly by E18R-42/PCDA vesicles. It was computed from the proportion diagnosed correctly by E18R-42/PCDA vesicles of the fourfold table given in Table 2. Chi-square test indicated that, there was no statistically significant difference (*p*>0.05) for the detection of *E*. *coli* O157:H7 with the standard culture method and our E18R-42/PCDA vesicles. It was thus clear that our aptasensor was feasible and reliable for primary screening *E*. *coli* O157:H7 in clinical fecal samples.

**Table 2:** Detection of *E*. *coli* O157:H7 in fecal samples by aptasensor and standard culture method.

E18R-42/PCDA vesicles	Standard culture method		Total
	+	–	
+	2	3	5
–	0	198	198
Total	2	201	203

*+*positive result;

–negative result.

In conclusion, we present a new aptasensor for rapid colorimetric detection of *E*. *coli* O157:H7. Through the experimental evidence, the mechanism behind the color change via TEM were elucidated. All these meant the aptasensor acted as both the molecular recognition element and the signal transducer element of the biosensor. This direct *E*. *coli* O157:H7 detection methodology relied on unique properties of PDA and aptamer, offers advantages rather than the conventional *E. coli* O157:H7 detection assays or other rapid detection methods. First, PDA vesicles and aptamers can be readily synthesized chemically with large quantities. Second, the aptasensor exhibits high specificity since aptamers could form complex secondary and tertiary structure for *E. coli* O157:H7. Also, the truncating on aptamers can cause them to increase their affinity to *E*. *coli* O157:H7 and reduce the cost for assays. Third, the assay took only 2 hours in time and 50 µl in volume, which saved time and volume and was very important especially at the time of encountering the wide spread of the disease. Last, by using this novel sensor, the results could be read by naked eye and not require any power or instrumentation. We expect that the aptasensor presented here provides a promising approach for pathogenic bacteria detection, and this method could be used not only in clinical analysis but also in biological terrorism defense.

## Materials and Methods

### Apparatus Materials

#### Bacteria

*E*. *coli O157: H7* was purchased from China Center of Industrial Culture Collection. *E. coli* (ATCC25922, CMCC44825, CMCC44155, CMCC44151), *Salmonella typhimurium* (CMCC50115), *Salmonella typhi* (CMCC50071), *Salmonella paratyphi* A(CMCC50433), *Salmonella paratyphi* B (CMCC50004), *Shigella sonnei* (*ATCC*25931), *Shigella flexneri* (CMCC51571), *Proteus vulgaris* (CMCC49072), *Enterobacter aerogenes* (ATCC 13048), *Citrobacter freundii* (ATCC8090), *Staphylococcus aureus* (ATCC25923) and *Monilia albican* (ATCC14053) were obtained from Wenzhou Medical College.

#### Reagent

PCDA was purchased from GFS chemicals. (Powell, OH, USA). 1,2-Dimyristoyl-sn-glycero-3-phosphocholine (DMPC), NHS and 1-Ethyl-3-(3-dimethylaminopropyl)carbodiimide, hydrochloride (EDC·HCl) were purchased from Sigma-Aldrich. (St Louis, MO). DNA oligonucleotides modified with amine group or FITC at the 5′ end were obtained from Bioneer Co. Ltd. (Shanghai, China). All solvents used in this study were of analytical grade and were prepared by using deionized water of resistivity not less than 18.2 MΩ cm.

#### Apparatus

PURELAB Classic was purchased from ELGA Lab Water (High Wycombe, UK). Scientz-IID ultrasonic homogenizer and SCIENTZ03-II UV crosslinker were purchased from Ningbo Scientz Biotechnology Co.Ltd. (Ningbo, China). CLSM Fluoview FV1000 was purchased from Olympus (Tokyo, Japan). TEM H-7500 was purchased from Hitachi (Tokyo, Japan). Varioskan Flash spectral scan multimode plate reader was purchased from Thermo Fisher Scientific (Waltham, MA, USA).

### PDA Vesicles Preparation and Conjugation

PCDA monomers were dissolved in chloroform and the solvent was removed by purging with nitrogen to generate a thin lipid film on the glass surface, and continued to be dried by vacuum for 2 hours. After adding the deionized water (18.2 MΩ cm) to make the 1 mM lipids, the lipids were sonicated at 72°C for 15 min until it turned clarity. The lipids were cooled in the dark bottle at 4°C overnight. They were polymerized by using UV light irradiation at 254 nm until turning blue, and then the vesicle solution was obtained. The quality of vesicle solution was monitored with Varioskan Flash spectral scan multimode plate reader and TEM. Most of the procedures for preparing LPS/PCDA/DMPC vesicle or PCDA/DMPC vesicle were the same as pure PCDA vesicle’s, excepting for replacing the PCDA monomer by a mixture containing PCDA monomer, DMPC and LPS (molar ratios as 6∶2: 0.1), or a mixture containing PCDA monomer and DMPC (molar ratios as 6∶2) to a total lipid concentration of 1 mM.

The aptamers were conjugated to the pure PCDA vesicles by using the carbodiimide method in the presence of the EDC and the NHS (Figure 1). Briefly, 6.25 µl of NHS (4 mM), 6.25 µl of EDC (4 mM) and 50 µl of pure PCDA (1 mM) vesicle were mixed in 187.5 µl of deionized water at room temperature. After stirring the mixtures for 2 h to active carboxy, 10 µl (0.5 mM) DNA (E17F-37, E18R-42 or random DNA) were added into the mixed solution. After incubating the mixtures at the room temperature overnight, the aptamers or random DNA were conjugated to the pure PCDA vesicles. Unbound aptamers were removed by dialysis with excess deionized water. The final volume was adjusted to 0.5 ml by adding deionized water and PCDA concentration was approximately 0.1 mM.

### Bacteria Preparation

Bacteria were cultured in the Luria Broth at 37°C overnight, and then washed 3 times with deionized water. Then appropriate 10-fold serial dilutions (10^−1^, 10^−2^, 10^−3,^ 10^−4^, 10^−5^, 10^−6^, 10^−7^ and 10^−8^) of the bacterial suspension were prepared in saline (0.9%). A 0.1 ml of each dilution was spread plated on to Luria Broth agar plate and the plates were incubated overnight at 37°C for 24 h. Finally, we got the *E. coli* O157:H7 samples of different concentrations from 10^3^ to 10^8^ CFU/ml via suspending bacteria with different volume deionized water.

### LPS-binding Aptamers Truncating and Affinity Test

As shown in table 3, the chosen aptamers were based on the sequences of E17F-72 and E18R-72 which could specifically bind to LPS of the *E. coli* O157:H7 [32]. With the purposes of making the aptamers work better and reducing the cost, the E17F-72 and the E18R-72 were truncated by deleting some sequences to investigate the relationships between structure and function. We finally got two aptamers, named E17F-37 and E18R-42 (Table 3). The sequences of these two pairs were loaded to the software of RNAstructure 4.5 and website of RNAfold to predict the second structures. The results indicated that they have the same loop in the second structures, implying the similar functions of them. CLSM was used to verify this implication. 5 µl (0.1 mM) different FITC-tagged aptamers (E17F-72, E17F-37, E18R-42 or E18R-72) were incubated with *E*. *coli* O157: H7 in the dark bottle at room temperature overnight. Then, the mixtures were purified by centrifugation at 5000 rpm and 3–4 times washes with 1 ml of 10 mM PBS buffer (pH 7.2). The mixtures were redispersed finally in the same buffer (10 µl) and placed onto a glass slide. After complete drying, the slides covered with coverslips were observed under CLSM equipped with an excitation wavelength of 488 nm and an emission wavelength of 520 nm.

**Table 3:** The sequence of LPS-binding aptamers and random DNA.

DNA	Sequences(5′-3′)	Length	source
E17F-72	ATCCGTCACACCTGCTCTATCAAATGTGCAGATATCA AGACGATTTGTACAAGATGGTGTTGGCTCCCGTAT	72 bp	[32]
E17F-37	ATCAAATGTGCAGATATCAAGACGATTTGTACAAGAT	37 bp	This study
E18R-72	ATACGGGAGCCAACACCATTCTATCGTTCCGGACGCT TATGCCTTGCCATCTACAGAGCAGGTGTGACGGAT	72 bp	[32]
E18R-42	CCGGACGCTTATGCCTTGCCATCTACAGAGCAGGTGT GACGG	42 bp	This study
random	GCCGGCTCAGCATGACTAAGAAGGAAGTTATGTGGTGTTGGC	42 bp	This study

And then, the E17F-37 and the E18R-42 were modified with the amide groups at the 5′ region and were used to prepare for the aptamer/PCDA vesicles. We performed the affinity tests to verify the binding sites of the E17F-37 and the E18R-42 by mixing the aptamer/PCDA vesicles to the LPS/DMPC/PCDA vesicles and the DMPC/PCDA vesicles. The DMPC/PCDA vesicles were used as the negative control. The results were observed by transmission electron microscopy (TEM).

### Characteristic Absorption Spectrum of Vesicles

The absorbance was obtained by using the Varioskan Flash spectral scan multimode plate reader from 228 nm to 700 nm at room temperature.

### Colorimetric Assays for *E. coli* O157: H7 and Sensitivity Test

Sensitivity test was performed as follows: the aptamer/PCDA vesicles were incubated with various concentrations of *E. coli* O157: H7 (10^3^ to 10^8^ CFU/ml) at 37°C for 2 hours. Absorbance measurements were performed at different time points using absorption spectroscopy from 400 nm to 700 nm. The aptamers of E17F-37 and E18R-42 automatically folded to capture the *E*. *coli* O157:H7, which would change the structure of PCDA backbone, and then induced the color turning blue to red. To quantify the degree of color change, we used the following formula [33], [34]: CR% = [(PB_0_– PB_f_)/PB_0_]×100%. Where the PB  =  A_640 nm_/(A_640 nm_ + A_540 nm_); the PB_0_ was in the absence of *E. coli* O157:H7, while the PB_f_ was in the presense of *E*. *coli* O157:H7. The higher CR% represents more efficient transition from the blue color to the red color.The data of A640 and A540 was collected before and after adding the O157:H7 from at least three independent experiments; the CR% values were then calculated.

### Specificity Testing

Specificity test was performed as follows: the aptasensors were used to detect other different bacteria at the concentration of 10^8^ CFU/ml but *E*. *coli* O157:H7 at the concentration of 10^7^ CFU/ml. The bacteria were as follows: *E. coli* (ATCC 25922, CMCC44825, CMCC44155, CMCC44151), *Salmonella typhimurium*, *Salmonella typhi, Salmonella paratyphi* A, *Salmonella paratyphi* B, *Shigella sonnei*, *Shigella flexneri*, *Proteus vulgaris*, *Enterobacter aerogenes*, *Citrobacter freundii*, *Staphylococcus aureus*, *Monilia albican* and *E*. *coli* O157: H7.

### Detection of *E. coli* O157:H7 in Fecal Samples

The clinical fecal specimens (*n* = 203), that were used for detecting *E*. *coli* O157:H7 by standard culture method and our E18R-42/PCDA vesicles, were from patients from the First Affiliated Hospital of Wenzhou Medical College. For artificially contaminated fecal samples, 1 ml of 10^8^ CFU/ml *E*. *coli* O157:H7 was added to each 1 g of fecal samples (*n* = 100) that were culture negative for *E*. *coli* O157:H7. For uncontaminated fecal samples, 1 ml of 10 mM sterile PBS buffer (pH 7.2) was added to each 1 g of fecal samples (*n* = 100) that were culture negative for *E*. *coli* O157:H7. All three kinds of samples need to be pretreated simply before application of E18R-42/PCDA vesicles. 1 g of fecal samples was homogenized in 8 ml of 10 mM sterile PBS buffer (pH 7.2) and was centrifuged at 500 rpm for 10 min. Then the supernatant was filtered through a 0.45 µm filtration device. Agitate the filter membrane carefully with 8 ml of sterile water in order to resuspend any bacteria that were caught in the filter membrane. Finally, bacteria were centrifuged at 12000 rpm for 10 min and resuspended in 1 ml of sterile water.

## Supporting Information

Figure S1 Negative-stained TEM images of PDA vesicles. (A) pure PCDA vesicles. (B) aptamer/PCDA vesicles. (C) DMPC/PCDA vesicles. (D) LPS/DMPC/PCDA vesicles. Scale bar is 100 nm.
(TIF)

Figure S2 CLSM images of affinity tests. (A) FITC labeled E17F-37 with *E*.*coli* O157:H7. (B) FITC labeled E18R-42 with *E*.*coli* O157:H7. (C) FITC labeled E17F-37 with *Salmonella typhimurium*. (D) FITC labeled E17F-72 with *E*.*coli* O157:H7. (E) FITC labeled E18R-72 with *E*.*coli* O157:H7. (F) FITC labeled E18R-42 with *Salmonella typhimurium*.
(TIF)
