# Correction: An Aptamer-Based Biosensor for Colorimetric Detection of *Escherichia coli* O157:H7

**DOI:** 10.1371/annotation/c8ed61a9-dcbf-4e20-8525-9da07ec92517

**Published:** 2013-05-10

**Authors:** Wenhe Wu, Jie Zhang, Meiqin Zheng, Yuhong Zhong, Jie Yang, Yuhong Zhao, Wenping Wu, Wei Ye, Jie Wen, Qi Wang, Jianxin Lu

There was an error in the Funding statement.

The correct Funding statement is: This study was supported by the National High Technology Research and Development Program of China (863) (2007AA022008), the National Natural Science Foundation of China (81101148), Zhejiang Provincial Program for the Cultivation of High-level Innovative Health talents and Key Laboratory of Laboratory Medicine, Ministry of Education, Key Science and Technology Innovation Team of Zhejiang (2010R50048), Zhejiang Province Science and Technology Planning Project (2009C33036), Major Project of Wenzhou science and Technology Foundation (Y20080080), Wenzhou Science and Technology Planning Project (Y20110021). The funders had no role in study design, data collection and analysis, decision to publish, or preparation of the manuscript. 

