# Supplementary figures and images for: An Aptamer-Based Biosensor for Colorimetric Detection of Escherichia coli O157:H7
Source: PLoS One. 2012 Nov 7;7(11):e48999. doi: 10.1371/journal.pone.0048999 (PMC3492178; doi:10.1371/journal.pone.0048999)

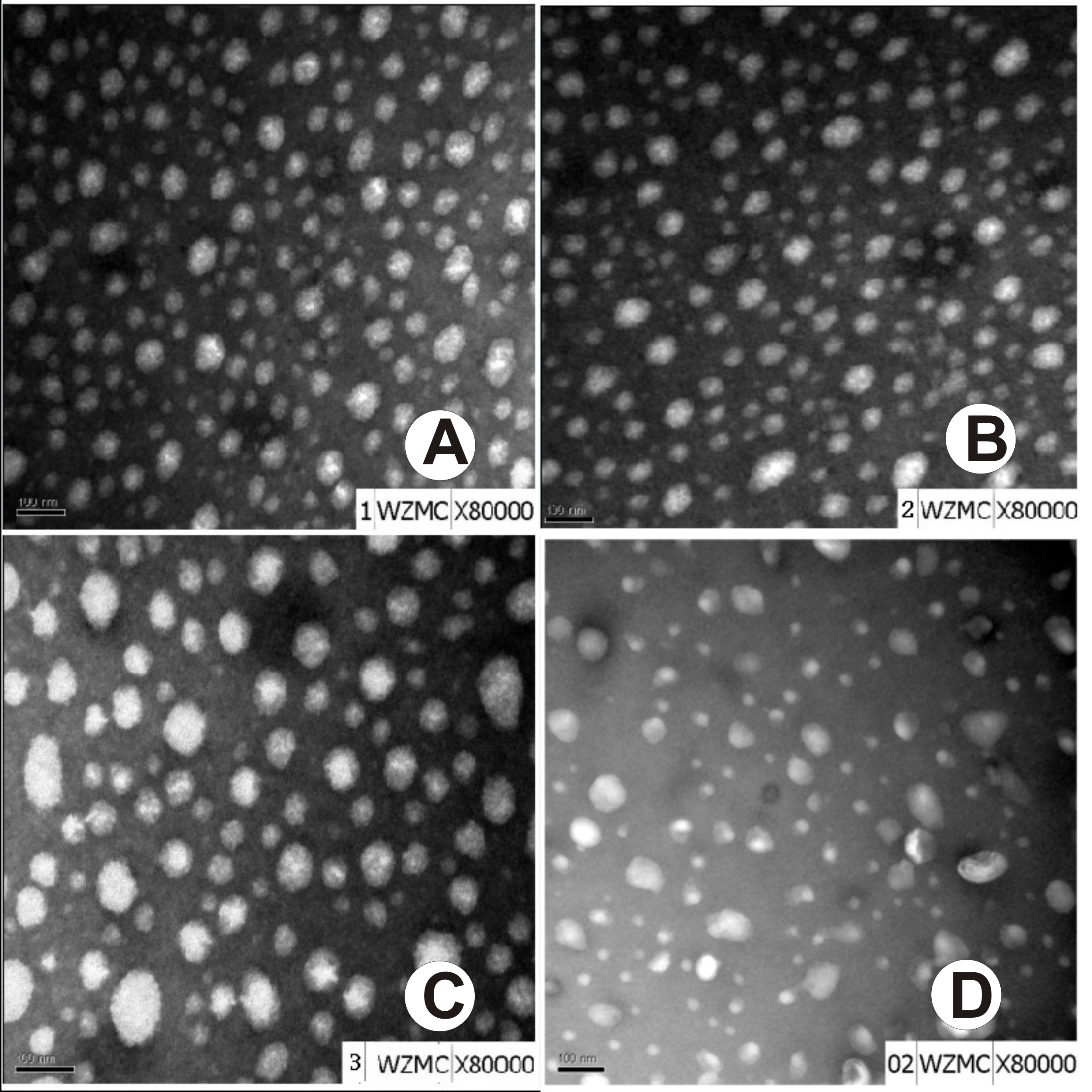

Supplement: Figure S1 — Negative-stained TEM images of PDA vesicles. (A) pure PCDA vesicles. (B) aptamer/PCDA vesicles. (C) DMPC/PCDA vesicles. (D) LPS/DMPC/PCDA vesicles. Scale bar is 100 nm. (TIF) [file pone.0048999.s001.tif]

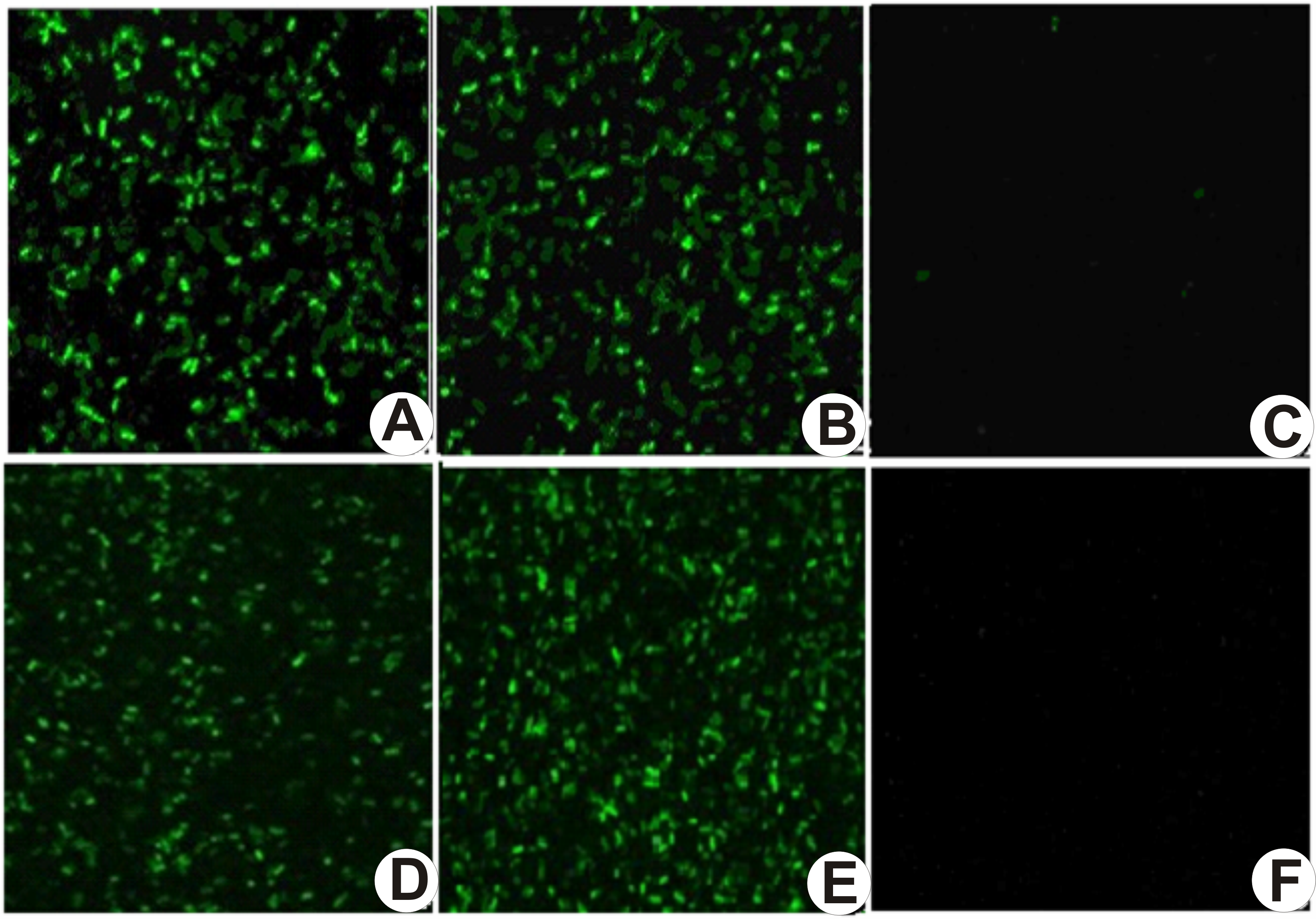

Supplement: Figure S2 — CLSM images of affinity tests. (A) FITC labeled E17F-37 with E.coli O157:H7. (B) FITC labeled E18R-42 with E.coli O157:H7. (C) FITC labeled E17F-37 with Salmonella typhimurium. (D) FITC labeled E17F-72 with E.coli O157:H7. (E) FITC labeled E18R-72 with E.coli O157:H7. (F) FITC labeled E18R-42 with Salmonella typhimurium. (TIF) [file pone.0048999.s002.tif]
